# The Effect of Hydrophobic Modified Block Copolymers on Water–Oil Interfacial Properties and the Demulsification of Crude Oil Emulsions

**DOI:** 10.3390/polym16172392

**Published:** 2024-08-23

**Authors:** Juan Zhang, Ping Liu, Yuan Gao, Qingping Yu

**Affiliations:** 1 State Key Laboratory of Shale Oil and Gas Enrichment Mechanisms and Effective Development, Beijing 102206, China; liuping.syky@sinopec.com; 2 Research and Development Center for the Sustainable Development of Continental Sandstone Mature Oilfield by National Energy Administration, Beijing 102206, China; 3 Unconventional Petroleum Research Institute, China University of Petroleum, Beijing 102249, China; gaoyuan@student.cup.edu.cn (Y.G.); 2023216607@student.cup.edu.cn (Q.Y.)

**Keywords:** block copolymer, hydrophobic modification, crude oil emulsion, demulsification, oil–water interfaces

## Abstract

The demulsification effect of three types of block copolymers, BP123, BPF123, and H123, with the same PEO and PPO segments but different hydrophobic modification groups on crude oil emulsions and the properties of oil–water interfaces were investigated using demulsification experiments, an interfacial tensiometer, and surface viscoelastic and zeta potential instruments in this paper. The results showed that the hydrophobic modification group of the block copolymers had great effects on the demulsification performance. The H123 block copolymers with the strongest hydrophobicity had the best demulsification effect on the crude oil emulsions. The properties of the oil–water interfaces indicated that the modified block copolymers achieved the demulsification of crude oil emulsions by reducing the strength of the oil–water interfacial film and the interfacial tension.

## 1. Introduction

The demulsification of crude oil emulsions is an essential issue in the petroleum industry. If crude oil contains water and impurities, the load of pumps, pipelines, and storage tanks will be increased during the gathering, transportation, and refining processes, and problems such as corrosion and the scaling of pipeline systems, pumps, and production equipment may be caused. In addition, oil in sewage will cause environmental pollution and will constitute a waste of resources [1,2]. Especially in the late stage of oil field development, the water content of the produced crude oil is becoming higher and higher, and the popularization and application of enhanced oil recovery technology may make the produced crude oil form oil-in-water or even multi-layer emulsions, which makes the demulsification of crude oil more difficult. Therefore, much attention has been paid to research on the demulsification of crude oil emulsions [3,4].

To break crude oil emulsions, various kinds of demulsification methods such as the physical method, the biological method, and the chemical method have been developed [5]. The physical method has some disadvantages, such as its complicated operation, high energy cost, low efficiency, long time, etc. [6]. The biological method only has good comprehensive performance of demulsification and dehydration for a certain crude oil emulsion [7]. The chemical demulsification method is a widely used means for the demulsification of crude oil emulsions, because the demulsifiers have the advantages of variable structures, rapid response, low cost, and high benefits and they can be applied to different types of emulsions [8,9]. The basic principle is to add a chemical demulsification agent to crude oil emulsions to destroy the emulsification state of crude oil and to separate oil from water. However, due to the complex composition of crude oil emulsions, the accurate mechanism still remains unknown. At present, the main demulsifiers used in oilfields are surfactants, polymers, ionic liquids, nanoparticles, etc. [10]. It is well known that polymer demulsifiers have played a crucial role over the years, especially the amphiphilic poly (ethylene oxide)–poly (propylene oxide) (PEO-PPO) block copolymers. 

In the past, the produced liquid from oilfields is usually treated separately, that is, W/O emulsions are demulsified with demulsifiers, and oily sewage is treated with cationic polymers. This step-by-step treatment method will not only lead to the loss of aging oil, but will also produce a large amount of sludge, which brings great problems to subsequent treatment [11]. Therefore, scientists have carried out lots of work on new synchronous treatment methods, such as by using multifunctional modified block copolymers, e.g., hydrophobic modified block copolymers, to simultaneously treat W/O emulsions and O/W emulsions, so as to synchronously achieve crude oil dehydration and sewage oil removal [12,13,14]. 

The PEO-PPO block copolymers, as low-molecular-weight surfactants, have a rich designability, which can be modified by changing the PEO/PPO ratio, chain length, branching degree, and molecular weight [15,16,17]. In terms of crude oil demulsification, PPO-PEO block copolymers have shown good application prospects [18]. There has been some studies on the demulsification of block copolymers on crude oil emulsions focusing on the composition and molecular structure of copolymers [19], the influence of inorganic salts [20,21,22], and the oil–water interface properties [23,24,25]. However, normal block copolymers cannot simultaneously treat W/O emulsions and O/W emulsions. 

Recently, investigations have shown that hydrophobic modifications can also have a great impact on the PEO-PPO copolymer properties, and much attention has been paid to the effect of the hydrophobic modification group on the interfacial properties of PEO-PPO copolymers. Gong et al. studied the air–water interface properties of PEO-PPO-ph-PPO-PEO and PPO-PEO-ph-PEO-PPO, and found that the copolymers with Bisphenol A molecules have a higher adsorption capacity and surface tension reduction efficiency compared with those without Bisphenol A molecules [26]. Sohn et al. studied the behavior of the hydrophobically modified polyethylene oxide at the air–water interface, and their studies show that the behavior of the hydrophobic ends anchored on the hydrophilic surface differs from the single hydrophobic amphiphilic molecule [27]. Liu et al. have synthesized three kinds of hydrophobic modified copolymers and found that with increasing hydrophobicity, the critical aggregation concentrations (CACs) and micelle size gradually decrease [28]. However, hydrophobic modified copolymers have barely been utilized in crude oil demulsification.

Therefore, this paper studied the influence of a series of block copolymers with the same PEO and PPO segments but different modified hydrophobic groups on the demulsification of both O/W and W/O emulsions. An interfacial tensimeter and an interfacial viscoelasticity meter were used to study the properties of the oil–water interface. The block copolymer H123, which had the strongest hydrophobicity, was proven to have the best demulsification effect on both O/W and W/O emulsions. This study may be useful for the petroleum science industry to achieve the simultaneous treatment of W/O and O/W emulsions, accordingly synchronously achieving crude oil dehydration and sewage oil removal.

## 2. Experimental

### 2.1. Materials

**Figure 1:**
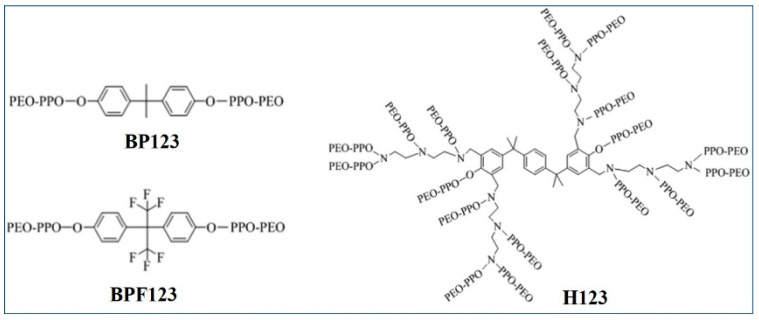
Molecular structure diagram of block copolymers BP123, BPF123, and H123.

The crude oil used here was obtained from Bamianhe Oilfield, with a density of 0.9215 g/cm^3^ (50 °C) and a viscosity of 232.0 mPa·s (50 °C). The kerosene used in preparing simulated oil was produced by Yanshan petrochemical company. In order to remove the impurities with interfacial activity, the kerosene was repeatedly treated via silica gel adsorption, and the oil–water interfacial tension reached more than 46 mN/m after treatment. Petroleum ether, magnesium chloride, calcium chloride, sodium bicarbonate, potassium chloride, magnesium sulfate, and anhydrous ethanol were all analytical grade. Sodium chloride was chemical grade. The ion composition of simulated formation water used in this study is given in Table 1. The modified block copolymers were gifts from Liu’s group [28] and their molecular structure diagram is given in Figure 1. Block copolymer Pluronic 123 (P123, PEO-PPO-PEO) was purchased from Sigma-Aldrich Co. (St. Louis, MO, USA). The molecular weight (M), PPO/PEO number ratio (n_PPO/PEO_), and the cloud point of the four copolymers are listed in Table 2.

### 2.2. Synthesis of Hydrophobic Modification Copolymers

The copolymers BP123, BPF123, and H123 were synthesized via anionic polymerization with different initiators and the modification ratio was controlled by the additive amount of the precursors, including bisphenol A, hexafluorobisphenol A, and phenamine resins. Firstly, the precursor, the potassium hydroxide, and the catalyst were added into a pressurized reactor under a N_2_ atmosphere with a stirrer. Then, propylene oxide was gradually added into the reactor by keeping the temperature at 120~140 °C and the pressure below 0.3 MPa. Then, ethylene oxide was added gradually. Finally, the product was obtained when the pressure no longer dropped and the temperature dropped to 90 °C. 

### 2.3. Preparation of Block Copolymer Solution

According to the actual production of the crude oil emulsion in the field, in order to better simulate binary combined flooding, a certain amount of polyacrylamide (300 mg∙L^−1^) and mahogany petroleum sulfonate (200 mg∙L^−1^) was dissolved in simulated water with stirring to prepare a 500 mg/L solution. The solution was stirred until completely dispersed and was then set aside for 24 h. A certain amount of block copolymers was dissolved in the above solution to obtain 1000 mg/L of demulsifier solution. 

### 2.4. Water-Containing Crude Oil Treatment

The crude oil was electrically dehydrated for 60 min with a DWY-1A multifunctional crude oil dehydration tester under a voltage of 380 V and a temperature of 60 °C to reduce the moisture content to less than 1 wt%. 

### 2.5. Preparation and Demulsification of O/W Emulsions

Simulated water was mixed with 0.2 wt% dehydrated crude oil and stirred using an IKA-T18 emulsifier at 1400 r/min for 5 min to obtain an O/W emulsion. Then, the emulsion was placed in a water bath at 25 °C for 4 h. The O/W emulsion was transferred from the beaker to a cylinder and mixed with the block copolymer solution. The mixture was shaken and immersed in a thermostatic water bath at 25 °C for 7 days to record the demulsification effect. 

### 2.6. Preparation and Demulsification of W/O Emulsions

W/O emulsions were obtained by mixing 30 wt% simulated water and 70 wt% dehydrated crude oil and stirring using an IKA-T18 emulsifier at 22,000 r/min for 5 min. Then, the block copolymer was mixed with the W/O emulsion and stirred at 6000 r/min for 1 min and immersed in a thermostatic water bath at 25 °C for 7 days. The water fraction was computed as V_t_/V_0_ × 100%, where V_0_ is the total water phase volume, and V_t_ is the volume of water phase measured after demulsification. 

### 2.7. Oil–Water Interfacial Tension Measurements

A JJ2000B spinning drop tensiometer was used to measure the interfacial tension between the simulated oil and the simulated formation water at 25 °C. 

### 2.8. Oil–Water Interfacial Shear Viscosity Measurements

The interfacial shear viscosity between the model oil and the simulated formation water was measured using an SVR.S interfacial shear viscosity meter made by Kyowa Scientific Co., Ltd. (Ofu City, Aichi Prefecture, Japan) at 25 °C. 

### 2.9. Microscope Image Test of Emulsions

The microscope images of emulsions were obtained using an Olympus BX41 microscope made by Olympus Corporation (Ishikawa, Hachioji City, Tokyo, Japan). 

### 2.10. Zeta Potential Measurements

The zeta potential of the O/W emulsions with block copolymer was measured the Delsa Nano Zeta potential and Nano particle size analyzer. In order to prevent the formation of a thick emulsion layer, which could affect the determination of Zeta potential, an appropriate amount of oil–water mixture was sheared at 14,000 r/min for 5 min, and was then mixed with the block copolymer. The mixture was stirred for 20 min. After being stored for 24 h, the zeta potential of the samples was measured at 25 °C. 

### 2.11. Oil Content Measurements

A total of 5 mL of demulsified O/W crude oil emulsion was placed in a separation funnel at a specific position in the middle of the test tube, and an appropriate amount of 1:1 hydrochloric acid was added; then, an appropriate amount of petroleum ether was added for extraction. The petroleum ether extract was placed in a 50 mL volumetric bottle, and the oil content was determined using ultraviolet spectrophotometry at a wavelength of 300 nm.

## 3. Results and Discussion

### 3.1. Demulsification Effect of Block Copolymers on O/W Emulsions

**Figure 2:**
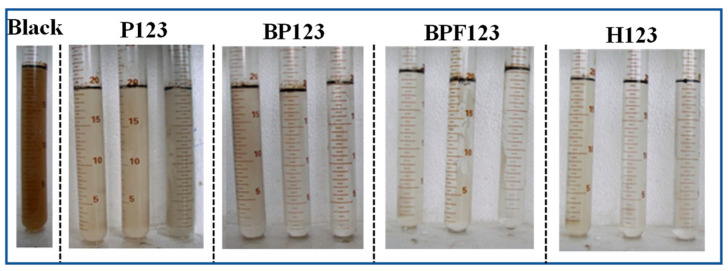
The samples of the O/W emulsions after demulsification by the four block copolymers. No copolymer was added in the sample labeled “Black”. The concentration of each block copolymer is 0, 5, 10, and 30 mg∙L^−1^ (from left to right).

The demulsification effect of the hydrophobic modified block copolymers on the O/W emulsions is shown in Figure 2, and the oil content of the samples after demulsification is shown in Table 3. The traditional block copolymer without hydrophobic modification, P123, with the same PPO/PEO number ratio as shown in Table 2 was also investigated as a comparison. It can be seen from Figure 2 that after adding the block copolymers, the solutions in the tubes became much clearer than that of the O/W emulsion samples without the block copolymer, which indicated that the copolymers had exerted a demusification effect on the O/W emulsions. The stronger the hydrophobicity of the copolymers, the better the demulsification effect on the O/W emulsions (H123 > BPF123 > BP123 > P123). In addition, it can also be seen from Figure 2 and Table 3 that with the increase in the modified block copolymer concentration, the demulsification effect of the O/W emulsion becomes better. In particular, the block copolymer H123 at a low concentration (5 mg/L) had shown very good demulsification effects. The further increase in concentration of H123 had no obvious effect on their demulsification ability for O/W emulsions. 

The zeta potential of the O/W emulsions with different kinds and concentrations of the block copolymers is shown in Table 4. Combined with the demulsification effect of the block copolymers in Table 3, it could be concluded that the more the absolute value of the zeta potential decreased after adding the block copolymers, the better the demulsification effect. The block copolymer H123 had the best demulsification effect on the O/W emulsion compared to the others, which mainly depended on the magnitude of the absolute zeta potential reduction on the surface of the oil droplets. The reason for the addition of block copolymers changing the zeta potential was that the ions destroyed the structure of water and increased the free water molecules in the system, so that the hydration effect of the modified copolymer molecules was enhanced [29]. Accordingly, the molecular conformation of the copolymers was more extended in order to grasp more small water droplets, and then the water droplets collided with each other and coalesced to form larger water droplets. That was to say, the presence of the ions was beneficial for the separation of the two phases of oil and water. 

**Figure 3:**
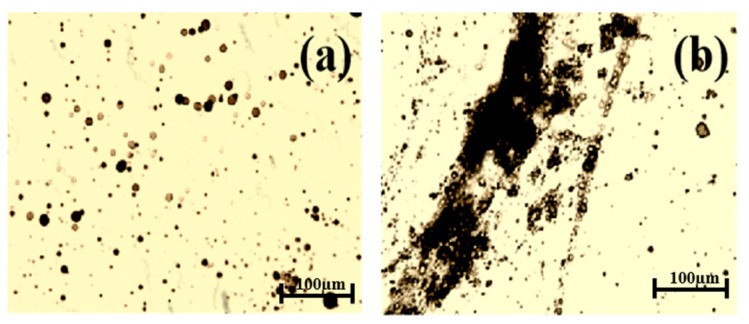
Microscope images of the O/W emulsion: (**a**) O/W emulsion without block copolymers and (**b**) O/W emulsion with 5 mg·L^−1^ H123.

Figure 3 shows the microscopic morphology changes in the O/W emulsion before and after adding 5 mg·L^−1^ H123. From Figure 3, it can be seen that in the O/W emulsion, the oil droplets were dispersed independently in water and their size was polydisperse (Figure 3a). After adding H123, some oil droplets gathered together and had a wide diameter distribution range (Figure 3b). That was, the stability of the O/W emulsion was destroyed after adding block copolymers. 

### 3.2. Demulsification Effect of Block Copolymers on W/O Emulsions

**Figure 4:**
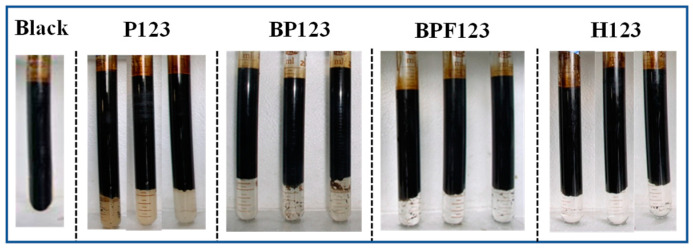
The samples of the W/O emulsions after demulsification by the four block copolymers. No copolymer was added in the sample labeled “Black”. The concentration of each block copolymer is 0, 5, 10, and 30 mg∙L^−1^ (from left to right).

Figure 4 displays the samples of the W/O emulsions after demulsification by the block copolymers; the corresponding water content is listed in Table 5. It can be seen from Figure 4 that without copolymers, the emulsion was stable. After the copolymers were added, the phenomenon of oil–water separation appeared in the emulsions. All the studied block copolymers had certain demulsification efficiencies, while the water quality and water separation rate were different. Specifically, in the W/O emulsions after demulsification by the block copolymers P123, BP123, and BPF123, the water was cloudy and still had some oil droplets. However, the H123 block copolymers had a better demulsification effect on W/O emulsions with a clear oil–water interface, and a clear effluent water and higher dewatering rate of crude oil emulsions, as revealed in Table 5. 

**Figure 5:**
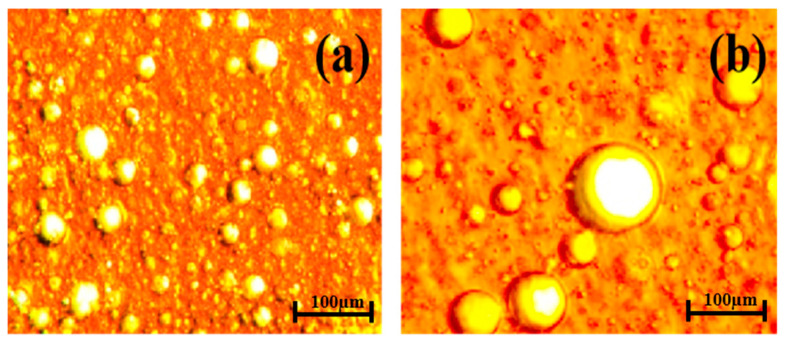
Electron microscope images of the W/O emulsion: (**a**) without block copolymers and (**b**) with 5 mg·L^−1^ H123.

Figure 5 shows the microscopic morphology changes in the W/O emulsion before and after adding 5 mg·L^−1^ H123. It can be seen from Figure 5a that in the W/O emulsion, the water droplets were dispersed independently in the oil, and their size was polydisperse with a range of a few microns. After adding the H123, as shown in Figure 5b, much larger water droplets with a size of dozens of microns were observed, indicating that the stability of the W/O emulsion was destroyed by the H123 molecules and the coalescence of the water droplets began to occur. 

The above results showed that the block copolymer H123 had a great demulsification effect on both W/O and O/W emulsions, which might be useful for the petroleum science industry to achieve the simultaneous treatment of W/O and O/W emulsions, also synchronously achieving crude oil dehydration and sewage oil removal. However, due to the good solubility of H123 in water, most of the H123 molecules still remain in the water after the demulsification of the crude oil emulsion. It is still a challenge to extract the H123 copolymers compared to other demulsifiers.

### 3.3. Interfacial Shear Viscosity between Oil and Block Copolymer Solution

**Figure 6:**
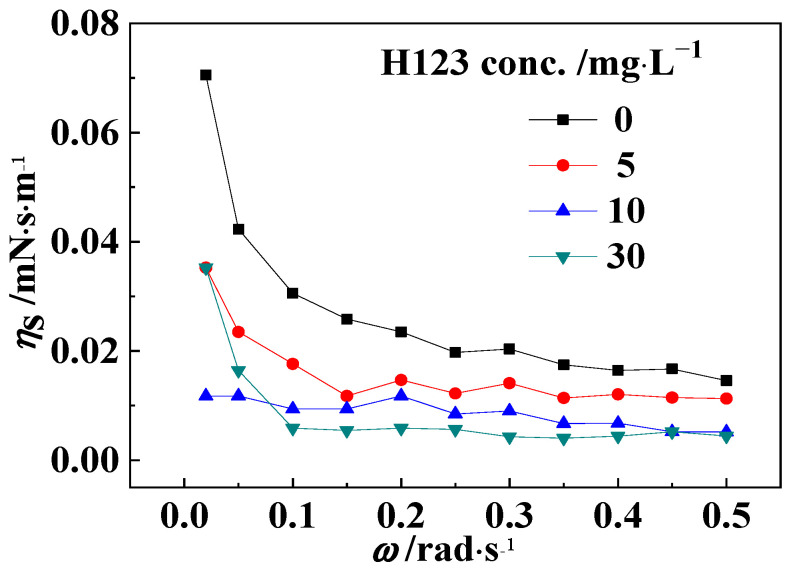
The interfacial shear viscosities of model oils and the H123 solutions with different concentrations.

For W/O or O/W emulsions, the emulsion stability is related to the intensity of the oil–water interfacial film, which can be characterized by the oil–water interfacial shear viscosity. Based on the above results, the polyether H123 was chosen to be studied. The interfacial shear viscosities of the H123 solutions with different concentrations and model oils are shown in Figure 6. The results clearly show that the oil–water interfacial shear viscosity obviously decreased after the addition of the H123 solution. This was mainly because the H123 molecules with a high interfacial activity could be adsorbed to the oil–water interface to replace the natural active components in crude oil, forming a new interfacial film with a lower strength. With the increase in the H123 solution concentration, the oil–water interfacial shear viscosity decreased gradually, which indicated that the interfacial film became weaker and easier to be destroyed. Combined with the above demulsification effect of the corresponding block copolymers, it could be inferred that the block copolymers could achieve demulsification by reducing the strength of the interface film between the oil and block copolymer solution. In addition, it was also seen that the oil–water interfacial shear viscosity decreased with increasing shear rate, and eventually remained unchanged when the concentration of the H123 solution was fixed. This was mainly attributed to the fact that after the shear rate was increased, the shear stress of the interfacial film increased to destroy the structure of the interfacial film, leading to a reduction in the shear viscosity of the interfacial film.

### 3.4. Interfacial Tension between Oil and Block Copolymer Solution

**Figure 7:**
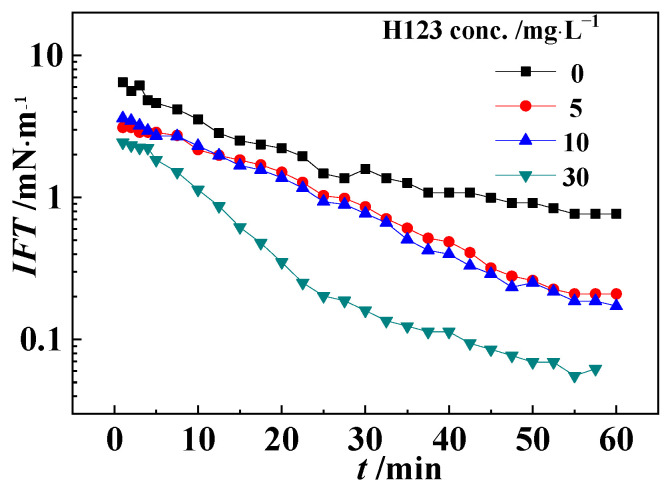
The interfacial tension of model oils and the H123 solutions with different concentrations.

The interfacial tension between the block polymer H123 solutions and the model oils are shown in Figure 7. The results show that the oil–water interfacial tension decreased obviously after adding the H123 polymer. This was mainly because the interfacial activity of H123 was higher than that of the interfacial active fractions of crude oil; therefore, the adsorption of the H123 molecules onto the oil–water interface resulted in a decrease in the interfacial tension, and eventually led to the demulsification of the crude oil emulsions. With H123 concentration increasing, the oil–water interfacial tension decreased as more H123 molecules were adsorbed on to the oil–water interface. The results were highly consistent with the oil–water interfacial shear viscosity results (Figure 6). 

### 3.5. The Mechanism of Demulsification

Based on the above results, it was found that the hydrophobic modified block copolymers BP123, BPF123, and H123 all showed a higher demulsification efficiency compared to P123 without hydrophobic groups. The stronger the hydrophobicity of the copolymers, the higher the demulsification efficiency of the crude oil emulsions (H123 > BPF123 > BP123). For one thing, the hydrophobic interaction between the hydrophobic groups of the copolymers and asphaltene molecules and the π-π interaction between the benzene rings of the copolymers and asphaltene molecules promoted the adsorption of the copolymers onto the oil–water interface. Accordingly, the hydrophobic modified copolymers had a stronger adsorption and interfacial activity than the asphaltene molecules; therefore, they could be preferentially adsorbed onto the oil–water interface to form a discontinuous mixed membrane with those un-desorbed asphaltene molecules. The strength and stability of the discontinuous mixed membrane was lower than that of the membrane formed by asphaltene, which resulted in the water beads coalescing to achieve demulsification [30]. For another thing, the branched copolymer H123 has a smaller vacancy than BP123 and BPF123 with straight chains; thus, it has a better ability to replace the film-forming material from the interface [31].

## 4. Conclusions

This paper demonstrated the demulsification effect of a series of hydrophobic modified block copolymers on crude oil emulsions and the properties of the crude oil–polyether solution interface. For O/W emulsions, the demulsification effect increased with the increase in the hydrophobicity and the concentration of the block copolymers. The block copolymers could adsorb on the oil–water interface to reduce the strength of the oil–water interface film and the interfacial tension. For W/O emulsions, all the studied block copolymers had obvious demulsification effects, while H123 showed the best effect as the water was clearest after demulsification. However, the mechanism of demulsification action is not completely clear and requires further study.

## Figures and Tables

**Table 1:** The components of the simulation water.

Ion	Cl^−^	SO_4_^2−^	HCO^−^_3_	Mg^2+^	Ca^2+^	Na^+^, K^+^
Concentration (mg/L)	3140.0	78.3	598.0	300	340	2430

**Table 2:** The properties of four block copolymers.

Polymers	P123	BP123	BPF123	H123
n_PPO/PEO_	1.75	1.76	1.76	1.76
M	5820	5980	6090	6510
Cloud point/°C	38	41	46	61

**Table 3:** Oil content of the samples after demulsification by the block copolymers.

Polymer Conc. (mg·L^−1^)	Oil Content (mg·L^−1^)				
	P123	BP123	BPF123	H123	Blank
5	523	261	19	6	768
10	302	192	10	4	
30	248	92	9	4	

**Table 4:** Zeta potential of the O/W emulsions after demulsification.

Polymer Conc. (mg·L^−1^)	ζ (mV)				
	P123	BP123	BPF123	H123	Blank
5	−30.28	−26.87	−5.80	−5.05	−43.24
10	−26.60	−21.69	−5.25	−4.86	
30	−20.55	−20.83	−5.15	−4.77	

**Table 5:** Water fraction of the W/O emulsion after demulsification by block copolymers.

Polymer Conc. (mg·L^−1^)	Dewatering Rate (%)				
	P123	BP123	BPF123	H123	Blank
5	60	65	80	88	0
10	65	70	85	95	
30	66	72	89	97	

## Data Availability

The original contributions presented in the study are included in the article, further inquiries can be directed to the corresponding author.

## References

[1] Xia L.X., Lu S.W., Cao G. (2004). Salt-assisted microwave demulsification. Chem. Eng. Commun..

[2] Wu X. (2002). Investigating the Stability Mechanism of Water-in-Diluted Bitumen Emulsions through Isolation and Characterization of the Stabilizing Materials at the Interface. Energy Fuels.

[3] Xia L., Lu S., Cao G. (2004). Stability and demulsification of emulsions stabilized by asphaltenes or resins. J. Colloid Interface Sci..

[4] Kang W.L., Liu S.R., Xu B., Wang X.Z., Zhang B.T., Bai B.J. (2013). Study on Demulsification of a Demulsifier at Low Temperature and Its Field Application. Pet. Sci. Technol..

[5] Yonguep E., Kapiamba K.F., Kabamba K.J., Chowdhury M. (2022). Formation, stabilization and chemical demulsification of crude oil-in-water emulsions: A review. Pet. Res..

[6] Dhandhi Y., Chaudhari R.K., Naiya T.K. (2022). Development in separation of oilfield emulsion toward green technology—A comprehensive review. Sep. Sci. Technol..

[7] Akbari N., Biria D. (2018). Investigation of the activity of Acinetobacter calcoaceticus biodemulsifier to break stable water in oil emulsions. J. Environ. Chem. Eng..

[8] Jabbari M., Izadmanesh Y., Ghavidel H. (2019). Synthesis of ionic liquids as novel emulsifier and demulsifiers. J. Mol. Liq..

[9] Yang X.G., Tan W., Tan X.F. (2009). Demulsification of Crude Oil Emulsion via Ultrasonic Chemical Method. Pet. Sci. Technol..

[10] Alara O.R., Abdurahman N.H., Tade M.O., Ali H.A., Alao K.T. (2022). Demulsifier: An important agent in breaking crude oil emulsions. Chem. Eng. Technol..

[11] Al-Sabagh A.M., Kandile N.G., Noor El-Din M.R. (2011). Functions of Demulsifiers in the Petroleum Industry. Sep. Sci. Technol..

[12] Sun L., Jiang H., Zhao Y.X., Deng X.Y., Ke S., Li Y., Tian M.G. (2021). Implementation of fluidized-bed Fenton as tertiary treatment of nitro-aromatic industrial wastewater. Process Saf. Environ. Prot..

[13] Otaibi A., Elkamel A., Al-Sahhaf T., Ahmed A.S. (2003). Experimental investigation of crude oil desalting and dehydration. Chem. Eng. Commun..

[14] Xu X.Z., Cao D., Liu J., Gao J., Wang X.Y. (2019). Research on ultrasound-assisted demulsification/dehydration for crude oil. Ultrason. Sonochemistry.

[15] Jiang W.M., Chen Y.M., Chen M.C., Liu X.L., Liu Y., Wang T.Y., Yang J. (2019). Removal of emulsified oil from polymer-flooding sewage by an integrated apparatus including EC and separation process. Sep. Purif. Technol..

[16] Duan M., He J., Li D.J., Wang X.J., Jing B., Xiong Y., Fang S.W. (2019). Synthesis of a novel copolymer of block polyether macromonomer and diallyldimethylammonium chloride and its reverse demulsification performance. J. Pet. Sci. Eng..

[17] Alexandridis P. (1997). Poly (ethylene oxide)/poly (propylene oxide) block copolymer surfactants. Curr. Opin. Colloid Interface Sci..

[18] Kadam Y., Bharatiya B., Hassan P.A., Verma G., Aswal V.K., Bahadur P. (2010). Effect of an amphiphilic diol (Surfynol) on the micellar characteristics of PEO–PPO–PEO block copolymers in aqueous solutions. Colloids Surf. A Physicochem. Eng. Asp..

[19] Wang J., Hu F.L., Li C.Q., Li J., Yang Y. (2010). Synthesis of dendritic polyether surfactants for demulsification. Sep. Purif. Technol..

[20] Wang C.Y., An S.G., Li Z.W., Chen H., Yan Z.H., Tan Y.B. (2021). Novel epigallocatechin gallate-based polyether surfactants: Synthesis, characterization and demulsification properties. Colloids Surf. A Physicochem. Eng. Asp..

[21] Watanabe K., Watanabe Y., Masuda K., Yang Z., Kaneshima A., Motoyama A., Shima T., Tsuchiya K., Sakai H. (2022). Key factor of sponge phase formation in commercial polyethoxylated nonionic surfactant/cosurfactant/water systems and its unique feature at interface. Colloids Surf. A Physicochem. Eng. Asp..

[22] Li Z., Shi Z., Zhao S., Yin S., Tan G., Jing B., Tan Y. (2016). Synthesis and Properties of a Novel Branched Polyether Surfactant. J. Surfactants Deterg..

[23] Zhang Z., Xu G.Y., Wang F., Dong S., Chen Y. (2005). Demulsification by amphiphilic dendrimer copolymers. J. Colloid Interface Sci..

[24] Zhang Z., Xu G.Y., Wang F., Dong S.L., Li Y.M. (2004). Characterization and demulsification of poly (ethylene oxide)-block-poly (propylene oxide)-block-poly (ethylene oxide) copolymers. J. Colloid Interface Sci..

[25] Jiang G.Q., Wei L., Li C.Y. (2012). Research on Demulsification Features of Polymer Flooding Produced Fluids of O/W with High Water Content for Oilfield and the Demulsification Efficacy. Res. J. Appl. Sci. Eng. Technol..

[26] Gong H., Xu G., Liu T., Xu L., Zhai X., Zhang J., Lv X. (2012). Aggregation behaviors of PEO-PPO-ph-PPO-PEO and PPO-PEO-ph-PEO-PPO at an air/water interface: Experimental study and molecular dynamics simulation. Langmuir.

[27] Paeng K., Choi J., Park Y., Sohn D. (2003). Temperature effect of hydrophobically modified polyethylene oxide at the air–water interface. Colloids Surf. A.

[28] Chang D., Du C., Liu J., Sun W., Su Y., Zang D., Liu T. (2022). Effect of hydrophobic modification of block copolymers on the self-assembly, drug encapsulation and release behavior. J. Mol. Liq..

[29] Tong K., Zhang Y., Chu P.K. (2013). Evaluation of calcium chloride for synergistic demulsification of super heavy oil wastewater. Colloids Surf. A.

[30] Abdelrahman O.E., Ahmed M.T., Hamad A.A. (2021). Synthesis and application of novel gemini pyridinium ionic liquids as demulsifiers for arabian heavy crude oil emulsions. Colloids Surf. A Physicochem. Eng. Asp..

[31] Sun T., Lu Z., Wang Y., Sui Z., Bo P., Li M., Yu J. (2002). Influence of demulsifiers of different structures on interfacial dilational properties of an oil–water interface containing surface-active fractions from crude oil. J. Colloid Interface Sci..

